# The effect of ERCC1 and ERCC2 gene polymorphysims on response to cisplatin based therapy in osteosarcoma patients

**DOI:** 10.1186/s12881-018-0627-4

**Published:** 2018-07-06

**Authors:** Hadeel Obiedat, Nasr Alrabadi, Eyad Sultan, Marwa Al Shatti, Malek Zihlif

**Affiliations:** 1 Department of Pharmacology, Faculty of Medicine, The University of Jordan, Amman, 11942 Jordan; 2 Department of Pharmacology, Faculty of medicine, Jordan University of Science and Technology (JUST), Irbid, 22110 Jordan; 3 Department of Pediatric Oncology, King Hussein Cancer Center (KHCC), Amman, Jordan; 4 Department of Pathology and Laboratory, King Hussein Cancer Center (KHCC), Amman, Jordan

**Keywords:** Pharmacogenomics, Cisplatin, Osteosarcoma, ERCC1, ERCC2

## Abstract

**Background:**

Cisplatin is one of the major drugs that used in the treatment of osteosarcoma. Cisplatin exerts its function by making cisplatin-DNA adducts culminating in cellular death. These adducts found to be repaired by nucleotide excision repair (NER) pathway. This study aimed to evaluate if polymorphisms in two main genes in the NER pathway, excision repair cross-complementing group 1 and 2 (ERCC1 and ERCC2) could affect the histological response to cisplatin based chemotherapy or clinical outcomes, particularly, event free survival (EFS) and overall survival (OS) rates.

**Method:**

ERCC1 (C118T (rs11615) and C8092A (rs3212986)) and ERCC2 (A751C (rs171140) and G312A (rs1799793)) polymorphisms were analysed in 44 patients with osteosarcoma, who were treated with cisplatin based neoadjuvant chemotherapy. DNA was extracted from patient’s formalin-fixed paraffin-embedded (FFPE) samples, patient’s genotypes were determined by using polymerase chain reaction-restriction fragment length polymorphism PCR-RFLP assay. The distribution of the patients’ genotype and the allele frequencies were reported. The association between each of these genotypes and many clinical and patho-histological parameters (e.g. EFS, OS and patho-histological response to treatment) was examined. The associations between gender, tumor location, presence of metastasis at diagnosis, histological subtypes, and type of neoadjuvant chemotherapy and between the histological response, EFS and OS rates were also examined.

**Results:**

This study revealed that there was a positive and significant association between ERCC1 C8092 A genotypes and median EFS rate in years; patients who were carriers of C allele (CC & CA) were found to have longer EFS rates than patients with AA genotype (*P* value = 0.006) and the median EFS rates were respectively as following: 2.04, 0.24 years. As well, both the presence of metastasis and the histological subtype at the time of diagnosis, were able to affect the EFS rate but not the OS. However, there was a positive correlation between OS rate and the patients’ primary response to treatment.

**Conclusions:**

Our results suggested that ERCC1 8092 C allele may play a role as a candidate prognostic marker in patients with osteosarcoma.

**Electronic supplementary material:**

The online version of this article (10.1186/s12881-018-0627-4) contains supplementary material, which is available to authorized users.

## Background

Osteosarcoma is the most common primary bone tumor occurring in children and adolescents [1]. The most effective treatment of osteosarcoma involves neoadjuvant therapy that includes the administration of cisplatin combined with doxorubicin and high dose methotrexate before surgical resection of the primary tumor, followed by adjuvant Chemotherapy [2]. However, the 5-year survival rate of this approach is 60–70% for patients with non-metastatic osteosarcoma of the extremities. Over the years these survival results have ceased even with the use of advanced chemotherapy [3, 4]. Chou and Gorlick (2006) illustrated the need to investigate the mechanisms of resistance to chemotherapy in osteosarcoma, and how it can be avoided [4].

Cisplatin is one of the most used chemotherapeutic agents in osteosarcoma treatment (reviewed in Majo et al., 2010) [5], which exerts it’s function by instigating DNA lesions through forming intrastrand and interstrand cross-links, which result in DNA distortion and cellular death [6]. The resistance to cisplatin may be intrinsic or acquired; tumor cells may gain this resistance by different mechanisms at DNA level or mechanisms that interrupt cisplatin binding to DNA which alternatively occurred at the plasma membrane or cytoplasmic level [7].

Several studies have illustrated numerous and multifocal mechanisms that involved in cisplatin resistance and can undermine the efficacy of treatment, such as reduction in cisplatin cellular accumulation, elevated levels of cisplatin deactivation by thiols, such as glutathione (GSH) and metallothionein (MT), enhanced DNA repair and changes in signal transduction pathways that participate in apoptosis [8, 9].

The DNA repair mechanism, in particular, the nucleotide excision repair (NER) pathway was found to be responsible for repairing cisplatin-bulky-DNA lesions [10]. Many studies suggested that defects in NER pathway may have a role in cancer onset [11, 12], as well as in the sensitivity to cisplatin chemotherapy [13–15].

Excision repair cross-complementing 1 (ERCC1) and excision repair cross-complementing 2 (ERCC2) are major members in NER pathway [16]. Many single nucleotide polymorphisms have been identified in ERCC1 and ERCC2 genes; of these polymorphisms, ERCC1 (C118T & C8092A) and ERCC2 (A751C & G312A) have been shown to affect the repair capacity and concomitantly the tumor’s sensitivity to cisplatin [17, 18].

The aim of this project is to elucidate the association between polymorphisms in ERCC1 (C118T & C8092A) and ERCC2 (A751C & G312A) genes and the response to cisplatin based chemotherapy. The effect of these SNPs on both, event free survival (EFS) and overall survival (OS) in patients with osteosarcoma, will be evaluated. As well, these findings can give indications for the genotype and the allele frequency of ERCC1 and ERCC2 SNPs in Arab population.

## Methods

### Study design

A retrospective observational cohort study was conducted on 44 patients who were diagnosed with primary osteosarcoma at King Hussain Cancer Center (KHCC) in the period between 2004 and 2012. The study protocol was approved by the institutional review board (IRB) of KHCC. Archived tissue samples from the tumors were obtained and used for DNA extraction. The patient’s demographic and clinical information were collected retrospectively from the hospital records.

### Cases selection criteria

In this study, patients treated with neoadjuvant chemotherapy containing cisplatin were included. The records, for all patients, reveal their histological response and clinical outcomes after treatment. Patients who didn’t have clear records or patients who were treated directly by tumor resection without neoadjuvant chemotherapy were excluded from the study.

### Genetic analysis

#### DNA extraction procedure

The DNA was extracted from archived formalin-fixed paraffin-embedded tissue samples. The extraction was obtained using QIAamp DNA FFPE Tissue Kit (Qiagen, Germany) according to the manufacturer’s recommendations with few modifications; in our study, the lysis time with proteinase K enzyme was increased to 26 h. In addition to that, 50 μl elusion buffer ATE was used with samples older than 5 years, while 70 μl was used for recent samples. The quality of DNA was checked using the Nano-Drop spectrophotometer and based on the ratio of absorbance at 260 and 280 nm. The values of the 260/280 ratio exceeded 1.6 for all used samples.

#### Genotyping

Polymerase chain reaction (PCR)–restriction fragment length polymerase (RFLP) assay was applied to detect ERCC1 and ERCC2 polymorphisms. The annealing temperature and the sequences of the primers which were used to amplify the targeted gene’s segments are shown in Table 1. PCR reactions were performed using a thermocycler (PTC-100TM Programmable thermal controller, MJ Research, Inc., USA). 2.5 μl DMSO was added to all reactions to reduce the non-specific bindings. A negative control lacking genomic DNA was included in each run of PCR. After running the samples on agarose gel, we usually got a smear and not a single genomic DNA band, which is common for FFPE samples. Instead of the DNA concentration estimated by the Nano-Drop, we used 10 μl of the DNA extract for all samples. However, if clear bands were not observed, then the reaction was repeated using 15 μl of the DNA sample. If the bands kept unseen after this adjustment, nested PCR was performed using 2 μl of regular PCR products instead of DNA extracts.

**Table 1:** Primers sequences and the expected product size

Gene of interest	Forward & reverse primers	Expected product size	Annealing T
ERCC1 C118T	F:5’GCAGAGCTCACCTGAGGAAC3’	208 bp (Won et al., 2010)	65 °C 1 min
	R:5’GAGGTGCAAGAAGAGGTGGA3’		
ERCC1 C8092A	F:5’CAGAGACAGTGCCCCAAGAG3’	161 bp (Zhou et al., 2004)	63 °C 45 s
	R:5’GGGCACCTTCAGCTTTCTTT3’		
ERCC2 G312A	F:5’CAGCTCATCTCTCCGCAGGATCAA3’	165 bp (Mechanic et al., 2005)	61 °C 45 s
	R:5’GTCGGGGCTCACCCTGCAGCACTTCCT3’		
ERCC2 A751C	F:5’TCAAACATCCTGTCCCTACT3’	344 bp (Sturgis et al., 2000)	60 °C 35 s
	R:5’CTGCGATTAAAGGCTGTGGA3’		

Analysis of patients’ genotypes were done using restriction enzymes, which recognize specific sequence in the DNA and digest at that sequence to give DNA fragments of precisely defined length. For ERCC1 118 C/T analysis, 3 μl of BsrDI enzyme (2000 u/ml) (NEB, Shanghai, China) were used to digest the PCR products of 208 bp. The reaction components were incubated for 4 h at 65 °C using PCR thermocycler. This reaction give two bands for TT genotype (128 bp and 80 bp), single band for CC genotype (208 bp) and three bands for CT genotype (208 bp, 128 bp and 80 bp). For ERCC1 8092 C/A, the PCR products of 161 bp were digested by 1 μl of MboII enzyme (5000 u/ml) (NEB, Shanghai, China). The reaction was done at 37 °C in incubator (MOD 2800) for 16 h. The restriction reaction gives two bands for AA genotype (101 bp and 60 bp), single band for CC genotype (161 bp) and three bands for CA genotype (161 bp, 101 bp and 60 bp). In case of ERCC2 G312A, the PCR products of 165 bp were digested for 16 h at 37 °C by StyI-HF™ enzyme (NEB, Shanghai, China). StyI-HF™ enzyme cuts only in the amplified fragment of the variant allele (A) to give two bands for AA genotype (139 bp and 26 bp), single band for GG genotype (165 bp) and three bands for GA genotype (165 bp, 139 bp and 26 bp). For ERCC2 A751C, PstI enzyme (NEB, Shanghai, China) was used to digest ERCC2 751 A/C PCR product of 344 bp for 16 h at 37 °C. The enzyme used has a single restriction site in the wild-type allele (A) resulting in two bands (234 and 110 bp), whereas the variant allele (C) results in three fragments (171, 110, and 63 bp). After RFLP reaction, the ERCC1 118 C/T and ERCC2 751 A/C digests were loaded onto 3.5% (*w*/*v*) agarose gel, while those for ERCC1 8092 C/A and ERCC2 312 G/A were loaded onto 4% (w/v) agarose gel. To detect the fragments, the gel visualized under an ultraviolet (UV) trans-illuminator (UVP Gel Doc-ItTM310 Imaging System, Upland, CA, USA) and photographed.

### Event free survival (EFS) and overall survival (OS) determination

Event free survival (EFS) time was calculated in years starting from the date of initiating chemotherapy and up to the date of the first event which the patient suffered from. This suffering event could be either development of metastasis, disease recurrence or even death. For those patients who did not faced any event, they were censored at the date of last follow up.

On the other hand, overall survival (OS) time was considered from the date of starting chemotherapy and up to the date of death. Patients, who were not deceased, were censored at the date of last follow up.

### Statistical analysis

For conducting univariate analysis related to the histological response, the Chi-square test was used for all descriptive analysis. Chi-square test was also used to analyze the association between histological response and all categorical factors like genotypes, alleles, gender, tumor location, presence of metastasis at diagnosis, histological subtypes and type of neoadjuvant chemotherapy.

Univariate analysis used to evaluate the associations between the previously mentioned factors and the histological response with OS and EFS were estimated by Kaplan-Meier method and were assessed by using log rank test. Cox’s regression hazard modelling of factors that were significant in univariate analysis was conducted to determine which factors could have a significant effect on OS and EFS. A probability level of 0.05 was used as the criterion of significance and all analysis were performed using SPSS (version 17.0).

## Results

### Patient characteristics

Patient’s demographic and clinical characteristics were collected retrospectively (Table 2), the oldest archived file in the study group was for a patient who was diagnosed in April 2004. The median age of the patients was approximately 14.7 years and the median follow up time after diagnosis was approximately 4.6 years.

**Table 2:** Patient’s demographics and clinical characteristics

Gender	Females, *N* = 17 (38.6%)
	Males, *N* = 27 (61.4%)
Age	Range 8.10–47.17 years
	Mean = 17.17 years
Grade	High grade, *N* = 34 (77.3%)
	Missing data, *N* = 10 (22.7%)
Histological subtypes	Conventional, *N* = 34 (89.5%)
	Variant, *N* = 4 (10.5%)
	Missed data, *N* = 6
Metastasis at diagnosis	Yes, *N* = 11 (25%)
	No, *N* = 33 (75%)
Neoadjuvant chemotherapy	MAP, *N* = 30 (68.2%)
	Cisplatin & Doxorubicin, *N* = 11 (25.0%)
	Other, *N* = 3 (6.80%)
Tumor location	Arm, *N* = 3 (6.8%)
	Femur, *N* = 22 (50%)
	Tibia, *N* = 15 (34.1%)
	Other, *N* = 4 (9.1%)
Response to chemotherapy	Good, *N* = 16 (36.4%)
	Poor, *N* = 28 (63.6%)

### Genotype frequencies for ERCC1 C118T, ERCC1 C8092A, ERCC2 G312A and ERCC2 A751C in the study group

The genotype frequencies in the study population for homozygous wild type, heterozygous and homozygous variant type, respectively, were as follows (Table 3): ERCC1C118T (27.3, 54.5 and 18.2), ERCC1 C8092A (29.55, 65.91 and 4.54), ERCC2 G312A (40.9, 52.3 and 6.8), and for ERCC2 A751C (46.3, 48.8 and 4.9).

**Table 3:** Genotypes frequencies in the studied group

ERCC1 C118T % (N)	ERCC1 C8092A % (N)	ERCC2 G312A % (N)	ERCC2 A751C % (N)
CC 27.3 (12)	CC 29.55 (13)	GG 40.9 (18)	AA 46.3 (19)
CT 54.5 (24)	CA 65.91 (29)	GA 52.3 (23)	AC 48.8 (20)
TT 18.2 (8)	AA 4.54 (2)	AA 6.8 (3)	CC 4.9 (2)
Total 44	Total 44	Total 44	Total 41

### Allele frequencies for ERCC1 (C118, 118 T, C8092 and 8092A) and ERCC2 (G312, 312A, A751 and 751C) in the study group

Allele frequencies were calculated by using Hardy-Weinberg equation (p2 + 2pq + q2 = 1). The allele frequencies in the study group for wild and variant alleles, respectively, were as follows (Table 4): ERCC1 C118T (54.55 and 45.45), ERCC1 C8092A (62.5 and 37.5), ERCC2 G312A (67.05 and 32.95), ERCC2 A751C (70.7 and 29.3).

**Table 4:** Alleles’ frequencies in the studied group

SNPs	Alleles carrier	Frequency % (N)
ERCC1 118 C/T	C allele	54.55 (48)
	T allele	45.45 (40)
ERCC1 8092 C/A	C allele	62.5 (55)
	A allele	37.5 (33)
ERCC2 312 G/A	G allele	67.05 (59)
	A allele	32.95 (29)
ERCC2 751 A/C	A allele	70.7 (58)
	C allele	29.3 (24)

### Univariate analysis for different variables with histological response

#### The ERCC1 and ERCC2 genotypes with the histological response

The associations between (ERCC1 C118T, ERCC1 C8092A, ERCC2 G312A and ERCC2 A751C) genotypes and the histological response were examined using Pearson chi-square test (Additional file 1: Table S1). All *p* values were more than 0.05; indicating that there is no statistically significant association.

#### ERCC1 and ERCC2 alleles with the histological response

The associations between (ERCC1 C118, ERCC1 118 T, ERCC1 C8092, ERCC1 8092A, ERCC2 G312, ERCC2 312A, ERCC2 A751 and ERCC2 751C) alleles and the histological response were tested by using Pearson chi-square test (Additional file 2: Table S2). Again none of the *p* values has reached the threshold for significant association (0.05).

#### Gender, tumor location, presence of metastasis at diagnosis, histological subtypes and types of neoadjuvant chemotherapy association with the histological response

The associations between gender, tumor location, presence of metastasis at diagnosis and histological subtypes and type of neoadjuvant chemotherapy with the histological response were examined by using Pearson chi-square test and no statistically association were found (Additional file 3: Table S3).

### Univariate analysis for variables with event free survival (EFS) rate

#### ERCC1 and ERCC2 genotypes with median EFS rate

**Fig. 1:**
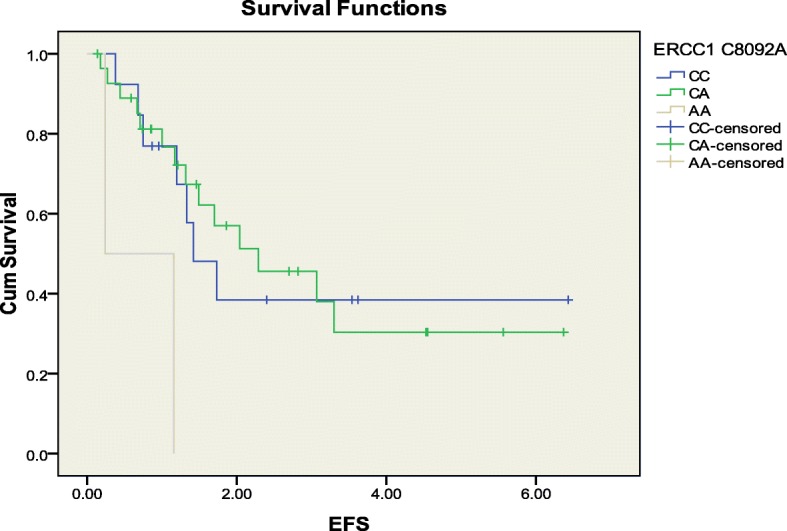
Kaplan-Meier plot of EFS (Years) according to ERCC1 C8092A polymorphism

The associations between ERCC1 C118T, ERCC1 C8092A, ERCC2 G312A, ERCC2 A751C genotypes with median EFS rate were examined using Kaplan-Meier/log rank method (Table 5). A strong association between ERCC1 C8092A genotypes (*p* value = 0.023) with median EFS rate of 1.42, 2.29, 0.24 for CC, CA and AA respectively (Table 5 & Fig. 1). While the median EFS rate did not vary significantly in patients with other genotypes (Table 5).

**Table 5:** Association between genotypes and median (EFS) rate in osteosarcoma patients treated with cisplatin combination

Genotypes	Median EFS survival rate in years	Log rank
ERCC1 118 CC	2.29	0.802
ERCC1 118 CT	1.42	
ERCC1 118 TT	1.73	
ERCC1 8092 CC	1.42	0.023
ERCC1 8092 CA	2.29	
ERCC1 8092 AA	0.24	
ERCC2 312 GG	1.42	0.147
ERCC2 312 GA	2.29	
ERCC2 312 AA	1.17	
ERCC2 751 AA	1.3	0.295
ERCC2 751 AC	2.8	
ERCC2 751 CC	Not reached

#### ERCC1 and ERCC2 alleles with median EFS:

**Fig. 2:**
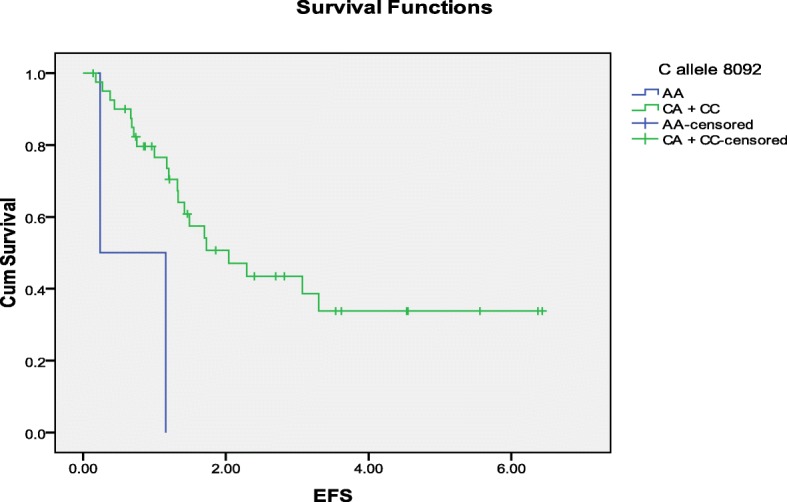
Kaplan-Meier plot of EFS (Years) according to ERCC1 8092 C allele carriers

The associations between ERCC1 C118, ERCC1 118 T, ERCC1 C8092, ERCC1 8092A, ERCC2 G312, ERCC2 312A, ERCC2 A751, ERCC2 751C alleles with median EFS rate were evaluated using Kaplan-Meier/log rank method (Table 6). A strong association was found between ERCC1 8092 C allele with median EFS rate of 2.04 and 0.24 for CC + CA, AA, respectively (*p* value = 0.006) (Table 8 & Fig. 2). A tendency toward association was found between ERCC2 312 G allele and median EFS rate of 2.04, 1.17 for GG + GA and AA, respectively (*p* value = 0.084) (Table 6).

**Table 6:** Association between Alleles and median EFS rate in osteosarcoma patients treated with cisplatin combinations

Alleles	Median EFS survival rate in years	Log rank
ERCC1 118 C allele	CC + CT 1.70	0.549
	TT 1.73	
ERCC1 118 T allele	TT + CT 1.49	0.647
	CC 2.29	
ERCC1 8092 C allele	CC + CA 2.04	0.006
	AA 0.24	
ERCC1 8092 A allele	AA+CA 2.04	0.879
	CC 1.42	
ERCC2 312 G allele	GG + GA 2.04	0.084
	AA 1.17	
ERCC2 312 A allele	AA+GA 2.04	0.511
	GG 1.42	
ERCC2 751 A allele	AA+AC 1.80	0.287
	CC not reached	
ERCC2 751 C allele	CC + AC 3.07	0.181
	AA 1.42

#### Gender, tumor location, presence of metastasis at diagnosis, histological subtypes and types of neoadjuvant chemotherapy association with median EFS rate

The associations between gender, tumor location, presence of metastasis at diagnosis and histological subtypes and type of neoadjuvant chemotherapy with median EFS rate were examined by using Kaplan-Meier/log rank method (Table 7). There was a tendency to association between presence of metastasis at diagnosis and median EFS rate (2.04, 0.75 for no metastasis and with metastasis respectively) with *p* value of 0.071. The results also showed a strong association in patients with conventional osteosarcoma (*p* value = 0.039) with median EFS rate of 2.04 years, while it was one year in patients with variant type of osteosarcoma.

**Table 7:** Association between different parameters and median EFS rate in osteosarcoma patients treated with cisplatin combinations

The studied parameter		Median EFS time in years	Log rank
Tumor location	Arm	1.49	0.477
	Femur	1.70	
	Tibia	2.29	
	Others	1.73	
Presence of metastasis	No metastasis	2.04	0.071
	With metastasis	0.750	
Gender	Male	2.04	0.289
	Female	1.16	
Histological subtypes	Conventional	2.04	0.039
	Variant	1.00	
Type of chemotherapy	MAP	1.73	0.432
	Cisplatin & doxorubicin	1.42	
	Others	3.07	
Response	Poor response	1.42	0.189
	Good response	3.07	

#### Cox regression analysis for EFS prognostic factors

After conducting multivariate analysis for ERCC1 8092 C allele, ERCC2 312 G allele, histological subtypes and presence of metastasis at diagnosis by using Cox regression hazard model, only ERCC1 8092 C allele and histological subtypes kept their significant association (Table 8).

**Table 8:** Factors associated with EFS rate

Variables	*P* value	Hazard ratio	95.0% CI
ERCC1 8092 C allele	0.044	59.008 (Reference =1)	1.122–3102.7
ERCC2 312 G allele	0.562	2.085 (Reference = 1)	0.174–24.955
Histology	0.026	0.149 (Reference = 1)	0.028–0.792
Presence of metastasis	0.078	0.406 (Reference = 1)	0.149–1.106

### Univariate analysis for variables with median OS rate

#### ERCC1 and ERCC2 genotypes with median OS rate

The associations between (ERCC1 C118T, ERCC1 C8092A, ERCC2 G312A and ERCC2 A751C) genotypes with median OS rate were examined by using Kaplan-Meier/log rank method (Additional file 4: Table S4). There was no significant association with *p* values more than 0.05.

#### ERCC1 and ERCC2 alleles with median OS rate

The associations between (ERCC1 C118, ERCC1 118 T, ERCC1 C8092, ERCC1 8092A, ERCC2 G312, ERCC2 312A, ERCC2 A751 and ERCC2 751C) alleles with median OS rate were evaluated by using Kaplan-Meier/log rank method (Additional file 5: Table S5). There was a tendency toward association between ERCC2 312 A allele and median OS rate, (not reached for AA+GA and 2.00 for GG) with *p* value of 0.058. While the median OS rate did not vary significantly in patients with other alleles, *p* value > 0.05.

#### Gender, tumor location, presence of metastasis at diagnosis, histological subtypes and types of neoadjuvant chemotherapy association with median OS rate

The associations between gender, tumor location, presence of metastasis at diagnosis and histological subtypes and type of neoadjuvant chemotherapy with median OS rate were examined by using Kaplan-Meier/log rank method (Additional file 6: Table S6). Interestingly, patients with good histological response had longer overall survival rate that differs significantly from those with poor histological response, with *p* value of 0.046.

#### Cox regression analysis for OS prognostic factors

After performing cox regression analysis, neither ERCC2 312 A allele nor the histological response remained the significant association in the model with *p* value > 0.05 (Additional file 7: Table S7).

## Discussion

Many studies have investigated the potential predictive and prognostic role of ERCC1 (C118T and C8092A) and ERCC2 (A751C and G312A) genetic polymorphisms on either tumors’ response to cisplatin chemotherapy or clinical outcomes [14, 17, 19–23]. Of these studies, many have shown significant associations with some of these variants, but others still revealed contradictory outcomes. The most important finding in this study is the significant association between ERCC1 C8092A genotypes and median EFS rate, which were 1.42, 2.29, and 0.24 years for CC, CA and AA variants, respectively. Longer EFS rate was significantly associated with both CC and CA compared with AA variant. The positive association between this variant and better OS rate in patient with osteosarcoma was previously reported. However, the association with EFS rate was not clearly investigated [17, 21–25].

This strong association can be related to the reduction in the Function of ERCC1 and so limited NER function. Such reduction in the repair capacity may exhibit more aggressive tumorgenecity, which in turn may affect the response to chemotherapeutics such as cisplatin. Such an explanation was also embraced by Zhou et al. (2004) [26], who linked the presence of the variant genotype with more aggressive tumor phenotype. Moreover, this explanation can also be supported by the finding of Chen et al. (1999) [11], who found that this SNP is strongly associated with the onset of adult glioma. Importantly, none of these studies was able to demonstrate genotype association with EFS. Studies on colorectal and ovarian patients have also failed to show such association [27, 28]. Interestingly, some studies reported opposite results [14, 29]. This variation in the findings can be attributed to the various demographic and clinical settings where cisplatin is employed (i.e. gender, age, type of tumor, stage and volume of tumor, presence of metastasis at diagnosis and type of chemotherapy), different targeted end points and the variation in the frequency of C8092A between the populations reprinted in these studies.

Away from this controversy, the association shown here between ERCC1 C8092A and EFS implies that the patients may be a candidate for further investigation toward using ERCC1 C8092A polymorphisms as a prognostic clinical biomarker, indicating better median EFS rate in osteosarcoma patients. However, ERCC1 C8092A SNP was not associated with the patho-histological response to chemotherapy or the OS rate, which was also found by other studies [20, 27, 28]. Of worth mentioning, the two patients with AA genotype were having unfavourable clinical characteristics; both were poor responders to chemotherapies, both suffered from progressed or relapsed disease in less than a year and both were having metastases.

Regarding the ERCC1 C118T SNP, it was not found to be associated with EFS nor OS rates. This lack of association with EFS or OS has also been reported by other groups [20, 30] and also in other types of tumors [15, 16, 26, 28, 31]. However, Hao et al. (2012) revealed that the ERCC1 118 variant genotype was associated with better EFS [30]. Many other studies agreed with their result as well [27, 32, 33]. In our study, we also could not find any association between C118T SNP and histological response to chemotherapy. This finding can also agree with studies conducted on other cancers; for instance, ovarian cancer patients [28] and non-small cell lung carcer (NSCLC) patients [15, 16, 31].

Our results also suggested no association between ERCC2 A751C and EFS rate in patients with osteosarcoma. This finding can be supported by three studies that were performed on patients with NSCLC [16, 31, 34]. However in many other studies including some studies conducted on osteosarcoma patients, the variant genotype or allele was found to have better survival outcomes [20, 27, 29, 30, 35]. By contrast, many studies have shown that patients with the variant allele or genotype possessed lower survival outcomes [13, 16, 19, 36, 37].

We also found that ERCC2 A751C SNP was not associated with the response to chemotherapy. Many studies reported the same result in patients with NSCLC [16, 31, 37]. However, three studies illustrated that patients with the ERCC2 751 polymorphic allele or genotype had a reduced response to chemotherapy in osteosarcoma and colorectal cancer patients [13, 16, 19], while other studies have shown the opposite results [27, 29].

An interesting finding in our study is that the ERCC2 312 G allele has a trend toward association with better EFS, but without reaching statistical significance. Similarly, in osteosarcoma patients it was illustrated that this polymorphism has a tendency toward association with lower EFS [19].

Osteosarcoma is one of the most common and aggressive bone tumours. It affects young individuals who need prolonged chemotherapeutic treatments, therefore, they may be exposed to severe and long term toxicities. The current treatment strategy depends on early surgical resection and aggressive chemotherapies [38–41]. Unfortunately, new strategies for treatment failed within the last 30 years and patients’ choices are restricted to better understanding the existing therapeutic modalities [38]. Pharmacogenomics is a new science which is excessively being studied in osteosarcoma patients either to predict the clinical course of the disease and its prognosis or to determine the best therapeutic approach for each patients. However, clear guidelines for a useful pharmacogenomics-guided therapeutic approach are yet to be elucidated [7, 17, 38]. Our study, in combination with other similar studies, is trying to build a bulk of pharmacogenomics knowledge to create a predictive model to determine the accurate clinical course and the best therapeutic options for these patients. Indeed, more studies are needed in this field which can include higher number of patients with more intensive pharmacogenomics analysis.

## Conclusions

The results out of this study, from patients with osteosarcoma treated with cisplatin based neoadjuvant chemotherapy, demonstrated positive association between ERCC1 8092 C allele carriers and better EFS rates in patients with osteosarcoma, suggesting that it may be a good prognostic factor. No associations were found between ERCC1 C118T and ERCC2 A751C SNPs with neither histological response to chemotherapy nor EFS and OS rates. Finally, larger sample size studies are needed to confirm these findings.

## Additional files

Additional file 1: **Table (S1).** ERCC1 and ERCC2 polymorphisms and histological Response in osteosarcoma patients treated with cisplatin combinations. (DOCX 12 kb)

Additional file 2: **Table (S2).** ERCC1 and ERCC2 alleles and histological response in osteosarcoma patients treated with cisplatin combination (DOCX 12 kb)

Additional file 3: **Table (S3).** Association between different parameters and histological response in osteosarcoma patients treated with cisplatin combinations. (DOCX 61 kb)

Additional file 4: **Table (S4).** Association between genotypes and median overall survival (OS) rate in osteosarcoma patients treated with cisplatin combination. (DOCX 11 kb)

Additional file 5: **Table (S5).** Association between Alleles and median OS rate in osteosarcoma patients treated with cisplatin combinations. (DOCX 12 kb)

Additional file 6: **Table (S6).** Association between different parameters and median OS rate in osteosarcoma patients treated with cisplatin combinations. (DOCX 51 kb)

Additional file 7: **Table (S7).** Factors associated with OS rate. (DOCX 11 kb)

## Abbreviations

EFS: Event free survival
ERCC1: Excision repair cross- complementing group 1
ERCC2: Excision repair cross- complementing group 2
FFPE: Formalin-fixed paraffin-embedded
GSH: Glutathione
MT: Metallothionein
NER: Nucleotide Excision Repair
NSCLC: Non-small cell lung carcer
OS: Overall survival
PCR-RFLP: Polymerase chain reaction-restriction fragment length polymorphism
