# Supplementary material for: The effect of ERCC1 and ERCC2 gene polymorphysims on response to cisplatin based therapy in osteosarcoma patients
Source: BMC Med Genet. 2018 Jul 6;19:112. doi: 10.1186/s12881-018-0627-4 (PMC6035436; doi:10.1186/s12881-018-0627-4)
Supplement: Supplementary file 1 — Table (S1). ERCC1 and ERCC2 polymorphisms and histological Response in osteosarcoma patients treated with cisplatin combinations. (DOCX 12 kb) [file 12881_2018_627_MOESM1_ESM.docx]

| **Genotypes** | **Poor responders % (N)** | **Good responders % (N)** | **Total % (N)** | ***P* value**  (Pearson Chi-square test) |
| --- | --- | --- | --- | --- |
| ERCC1 118 CC | 13.6 (6) | 13.6 (6) | 27.3 (12) | 0.229 |
| ERCC1 118 CT | 40.9 (18) | 13.6 (6) | 54.5 (24) |  |
| ERCC1 118 TT | 9.1 (4) | 9.1 (4) | 18.2 (8) |  |
| ERCC1 8092 CC | 22.7 (10) | 6.8 (3) | 29.5 (13) | 0.220 |
| ERCC1 8092 CA | 36.4 (16) | 29.5 (13) | 65.9 (29) |  |
| ERCC1 8092 AA | 4.5 (2) | 0 (0) | 4.5 (2) |  |
| ERCC2 312 GG | 27.3 (12) | 13.6 (6) | 40.9 (18) | 0.318 |
| ERCC2 312 GA | 29.5 (13) | 22.7 (10) | 52.3 (23) |  |
| ERCC2 312 AA | 6.8 (3) | 0 (0) | 6.8 (3) |  |
| ERCC2 751 AA | 31.7 (13) | 14.6 (6) | 46.3 (19) | 0.794 |
| ERCC2 751 AC | 29.3 (12) | 19.5 (8) | 48.8 (20) |  |
| ERCC2 751 CC | 2.4 (1) | 2.4 (1) | 4.9 (2) |  |
