# Supplementary material for: The effect of ERCC1 and ERCC2 gene polymorphysims on response to cisplatin based therapy in osteosarcoma patients
Source: BMC Med Genet. 2018 Jul 6;19:112. doi: 10.1186/s12881-018-0627-4 (PMC6035436; doi:10.1186/s12881-018-0627-4)
Supplement: Supplementary file 2 — Table (S2). ERCC1 and ERCC2 alleles and histological response in osteosarcoma patients treated with cisplatin combination (DOCX 12 kb) [file 12881_2018_627_MOESM2_ESM.docx]

| **SNPs** | **Alleles carrier** | **Poor responders**  **% (N)** | **Good responders**  **% (N)** | **Total % (N)** | **P value**  **(Pearson Chi-square test)** |
| --- | --- | --- | --- | --- | --- |
| ERCC1 118 C/T | C allele | CC+CT 54.5 (24)  TT 9.1 (4) | CC+CT 27.3 (12)  TT 9.1 (4) | 81.8 (36)  18.2 (8) | 0.375 |
|  | T allele | TT+CT 50.0 (22)  CC 13.6 (6) | TT+CT 22.7 (10)  CC 13.6 (6) | 72.7 (32)  27.3 (12) | 0.250 |
| ERCC1 8092 C/A | C allele | CC+CA 59.1 (26)  AA 4.5 (2) | CC+CA 36.4 (16)  AA 0.0 (0) | 95.5 (42)  4.5 (2) | 0.274 |
|  | A allele | AA+CA 40.9 (18)  CC 22.7 (10) | AA+CA 29.5 (13)  CC 6.8 (3) | 70.5 (31)  29.5 (13) | 0.235 |
| ERCC2 312 G/A | G allele | GG+GA 56.8 (25)  AA 6.8 (3) | GG+GA 36.4 (16)  AA 0.0 (0) | 93.2 (41)  6.8 (3) | 0.175 |
|  | A allele | AA+GA 36.4 (16)  GG 27.3 (12) | AA+GA 22.7 (10)  GG 13.6 (6) | 59.1 (26)  40.9 (18) | 0.728 |
| ERCC2 751 A/C | A allele | AA+AC 61 (25)  CC 2.4 (1) | AA+AC 34.1 (14)  CC 2.4 (1) | 95.1 (39)  4.9 (2) | 0.686 |
|  | C allele | CC+AC 31.7 (13)  AA 31.7 (13) | CC+AC 22 (9)  AA 14.6 (6) | 53.7 (22)  46.3 (19) | 0.536 |
