# Supplementary figures and images for: The effect of ERCC1 and ERCC2 gene polymorphysims on response to cisplatin based therapy in osteosarcoma patients
Source: BMC Med Genet. 2018 Jul 6;19:112. doi: 10.1186/s12881-018-0627-4 (PMC6035436; doi:10.1186/s12881-018-0627-4)

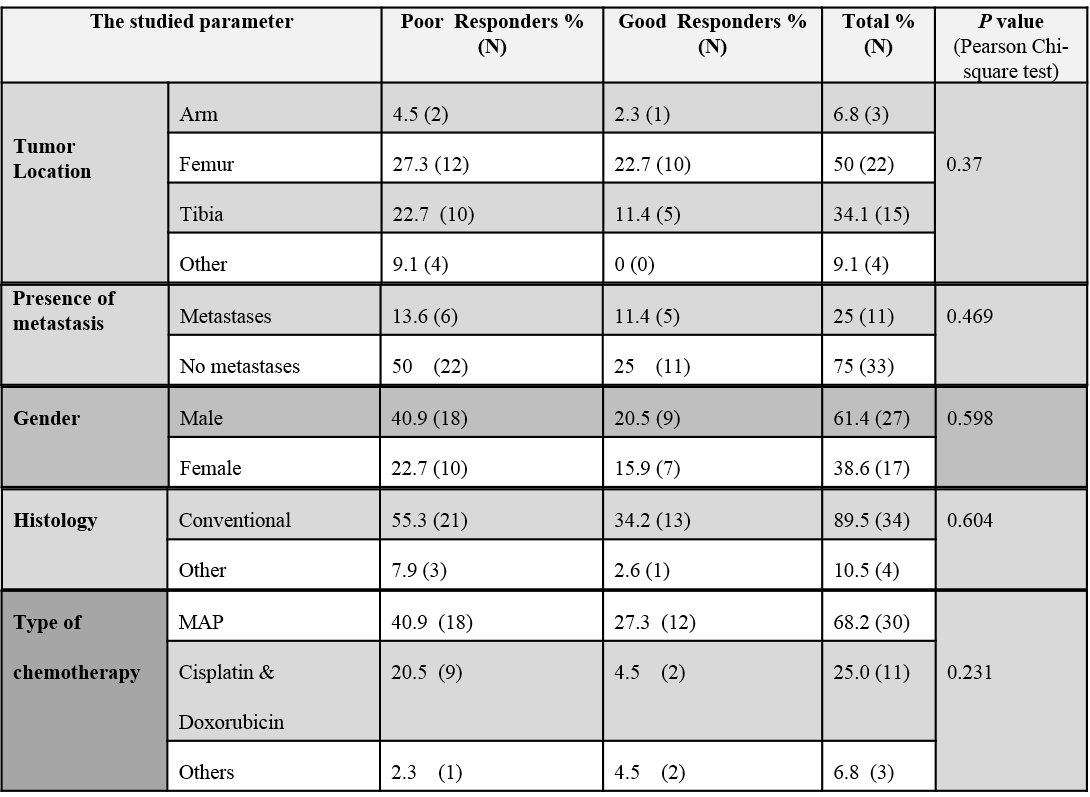

Supplement: Supplementary file 3 — Table (S3). Association between different parameters and histological response in osteosarcoma patients treated with cisplatin combinations. (DOCX 61 kb) [file 12881_2018_627_MOESM3_ESM.docx]

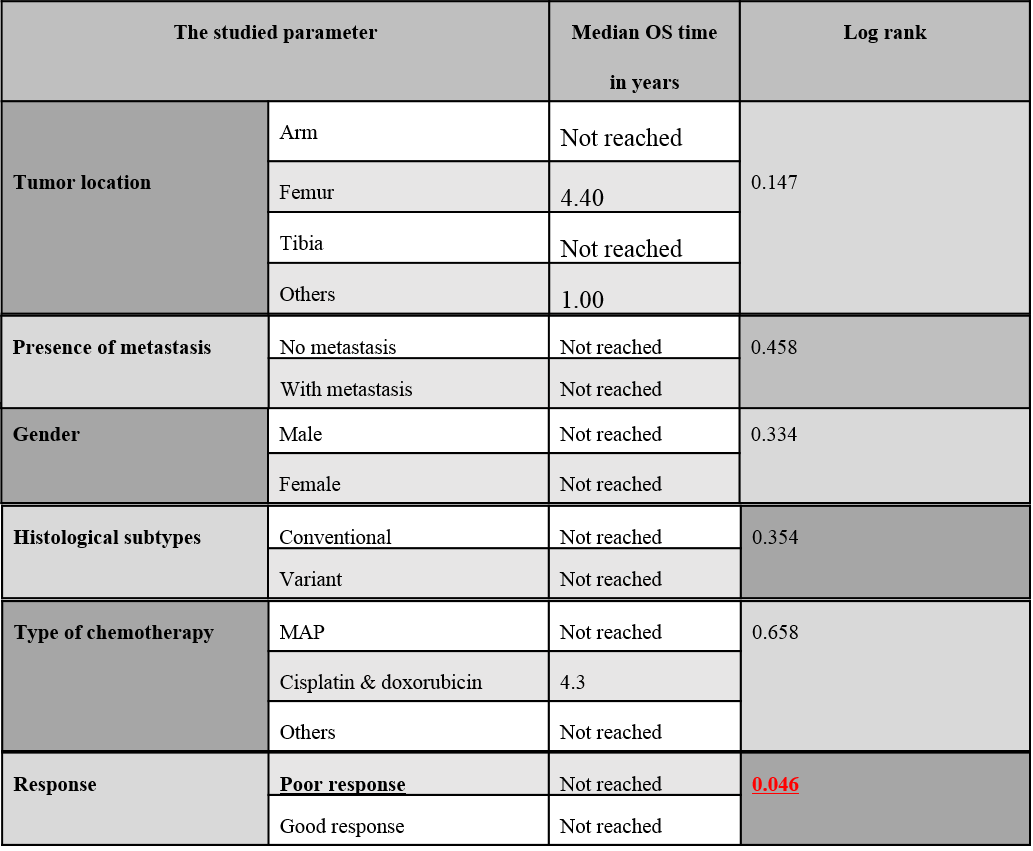

Supplement: Supplementary file 6 — Table (S6). Association between different parameters and median OS rate in osteosarcoma patients treated with cisplatin combinations. (DOCX 51 kb) [file 12881_2018_627_MOESM6_ESM.docx]
