# Supplementary material for: The effect of ERCC1 and ERCC2 gene polymorphysims on response to cisplatin based therapy in osteosarcoma patients
Source: BMC Med Genet. 2018 Jul 6;19:112. doi: 10.1186/s12881-018-0627-4 (PMC6035436; doi:10.1186/s12881-018-0627-4)
Supplement: Supplementary file 4 — Table (S4). Association between genotypes and median overall survival (OS) rate in osteosarcoma patients treated with cisplatin combination. (DOCX 11 kb) [file 12881_2018_627_MOESM4_ESM.docx]

| **Genotypes** | **Median OS survival rate in years** | **Log rank** |
| --- | --- | --- |
| ERCC1 118 CC | 4.40 | 0.536 |
| ERCC1 118 CT | Not reached |  |
| ERCC1 118 TT | Not reached |  |
| ERCC1 8092 CC | Not reached | 0.858 |
| ERCC1 8092 CA | Not reached |  |
| ERCC1 8092 AA | Not reached |  |
| ERCC2 312 GG | 1.80 | 0.164 |
| ERCC2 312 GA | Not reached |  |
| ERCC2 312 AA | Not reached |  |
| ERCC2 751 AA | Not reached | 0.808 |
| ERCC2 751 AC | Not reached |  |
| ERCC2 751 CC | Not reached |  |
