# Supplementary material for: The effect of ERCC1 and ERCC2 gene polymorphysims on response to cisplatin based therapy in osteosarcoma patients
Source: BMC Med Genet. 2018 Jul 6;19:112. doi: 10.1186/s12881-018-0627-4 (PMC6035436; doi:10.1186/s12881-018-0627-4)
Supplement: Supplementary file 5 — Table (S5). Association between Alleles and median OS rate in osteosarcoma patients treated with cisplatin combinations. (DOCX 12 kb) [file 12881_2018_627_MOESM5_ESM.docx]

| **Alleles** | **Median OS survival rate in years** | **Log rank** |
| --- | --- | --- |
| ERCC1 118 C allele | CC+CT not reached | 0.509 |
|  | TT not reached |  |
| ERCC1 118 T allele | TT+CT not reached | 0.50 |
|  | CC 4.40 |  |
| ERCC1 8092 C allele | CC+CA not reached | 0.685 |
|  | AA not reached |  |
| ERCC1 8092 A allele | AA+CA not reached | 0.74 |
|  | CC not reached |  |
| ERCC2 312 G allele | GG+GA not reached | 0.724 |
|  | AA not reached |  |
| ERCC2 312 A allele | AA+GA not reached | **0.058** |
|  | GG 2.00 |  |
| ERCC2 751 A allele | AA+AC not reached | 0.617 |
|  | CC not reached |  |
| ERCC2 751 C allele | CC+AC not reached | 0.748 |
|  | AA not reached |  |
