# Supplementary material for: The effect of ERCC1 and ERCC2 gene polymorphysims on response to cisplatin based therapy in osteosarcoma patients
Source: BMC Med Genet. 2018 Jul 6;19:112. doi: 10.1186/s12881-018-0627-4 (PMC6035436; doi:10.1186/s12881-018-0627-4)
Supplement: Supplementary file 7 — Table (S7). Factors associated with OS rate. (DOCX 11 kb) [file 12881_2018_627_MOESM7_ESM.docx]

| **Variables** | **P value** | **Hazard ratio** | **95.0% CI** |
| --- | --- | --- | --- |
| ERCC2 312 A allele | 0.149 | 2.907 (Reference = 1) | 0.683-12.366 |
| Histological response | 0.118 | 0.182 (Reference = 1) | 0.022-1.541 |
